# Post-transplant diabetes mellitus in kidney transplant recipients: insights from continuous glucose monitoring

**DOI:** 10.1093/ckj/sfag252

**Published:** 2026-08-25

**Authors:** Khaled Oweidat, Christopher K Farmer

**Affiliations:** Department of Renal Medicine, East Kent Hospitals University NHS Foundation Trust, Canterbury, Kent, UK; Kent and Medway Medical School, University of Kent and Canterbury Christ Church University, Canterbury, Kent, UK; Department of Renal Medicine, East Kent Hospitals University NHS Foundation Trust, Canterbury, Kent, UK; Centre for Health Services Studies, University of Kent, Canterbury, Kent, UK

To the Editor,

Post-transplant diabetes mellitus is a common metabolic complication of kidney transplantation, affecting ∼10%–30% of kidney transplant recipients and carrying implications for cardiovascular risk and graft survival [1]. Glycaemic assessment relies heavily on glycated haemoglobin (HbA1c), yet its accuracy is challenged in transplant recipients by altered erythrocyte kinetics, anaemia, and fluctuating renal function [1–3]. Continuous glucose monitoring (CGM) offers a more direct measure of glycaemic exposure, but its comparative reliability against conventional methods including the oral glucose tolerance test and HbA1c in this population remains uncharacterized, and CGM is not routinely incorporated into post-transplant care protocols.

We conducted a single-centre retrospective observational study at East Kent Hospitals University NHS Foundation Trust, United Kingdom, including 17 adult kidney transplant recipients with PTDM using FreeStyle Libre flash glucose monitoring. For each patient, available glucose data were divided into consecutive three-month periods, each aligned to an HbA1c measured at the end of that period; the number of periods contributed per patient varied with the duration of available monitoring (median 5 periods per patient; range 2–9), yielding 78 paired CGM–HbA1c observations across the cohort. CGM-derived metrics were compared with concurrent HbA1c. These comprised mean glucose; the glucose management indicator (GMI), an estimate of HbA1c calculated from the mean sensor glucose over the monitoring period and reported on the same scale (mmol/mol); time in range (TIR), the percentage of time glucose was within the target range of 3.9–10.0 mmol/l; and the coefficient of variation (CV), a measure of glycaemic variability. In contrast to laboratory HbA1c, which reflects the glycation of haemoglobin over the roughly 8–12-week red-cell lifespan and can be distorted by anaemia or altered erythrocyte turnover, the GMI is derived directly from the interstitial glucose measured by the CGM sensor [3]. Ethical approval was obtained; the study was conducted in accordance with the Declaration of Helsinki.

Median HbA1c was 57 mmol/mol (IQR 49–68) and median CGM-derived GMI was 55 mmol/mol (IQR 51–64), with median TIR of 64.0% (IQR 48.3–79.5). Despite similar central tendencies, marked HbA1c–GMI discordance (absolute difference ≥5 mmol/mol) was observed in 47.4% of periods; among these, discordance exceeded 10 mmol/mol in 17.9%. HbA1c and GMI therefore diverged at the individual-period level in nearly half of monitoring periods, indicating that, in the context of the transplant-specific confounders described above, HbA1c alone may under- or over-estimate glycaemic exposure in this cohort.

Substantial glycaemic variability persisted even in periods classified as controlled by HbA1c (<53 mmol/mol). Elevated CGM-derived measures of glycaemic variability were observed in 25–61% of these controlled periods. Given emerging evidence that glycaemic variability contributes to macrovascular risk [4], this hidden variability is particularly relevant in kidney transplant recipients, in whom cardiovascular disease remains the leading cause of mortality [5].

Our findings demonstrate that in kidney transplant recipients with PTDM, HbA1c incompletely reflects glycaemic exposure and variability. Marked HbA1c–GMI discordance and substantial hidden variability were observed in a considerable proportion of monitoring periods, indicating that HbA1c alone may underestimate the true glycaemic burden in this population. Integration of CGM into post-kidney transplant care may improve glycaemic assessment and support more individualized management. Larger prospective studies with extended CGM monitoring are warranted to characterize the drivers of discordance and to evaluate the impact of CGM-guided care on graft and cardiovascular outcomes.
